# The other pandemic: lessons from 40 years of HIV research

**DOI:** 10.1172/JCI183039

**Published:** 2024-07-01

**Authors:** Mary E. Klotman, Barton F. Haynes

**Affiliations:** 1 Department of Medicine,; 2 Department of Molecular Genetics and Microbiology,; 3 Department of Pathology,; 4 Integrative Immunobiology, and; 5 Duke Human Vaccine Institute, Duke University School of Medicine, Durham, North Carolina, USA.

Since 1983, the *JCI* has consistently published papers that have contributed to much of today’s understanding of the immunopathogenesis of HIV-1–host interactions. A representative subset of papers published in the *JCI* since the inception of the AIDS pandemic will be highlighted here. These reports have had a major impact on the understanding of pathogenesis and the clinical management of HIV-1 infection and have opened the door to current and future research, promising to better prevent, manage, and even cure HIV-1 infection.

## Functional immune abnormalities in AIDS

Early on in the AIDS pandemic and before identification of the etiologic virus, Landay and colleagues (1) as well as Nicholson et al. (2) demonstrated that a deficiency of CD4^+^ T cells and an inverted CD4:CD8 ratio were present in asymptomatic hemophiliac individuals and in men who have sex with men (MSM) with lymphadenopathy. These features were soon recognized as hallmarks of the profound immunodeficiency-associated infection. At the same time, teams from the NIH or the National Center for Drugs and Biologics contributed to our understanding of the full breadth of the immunodeficiency by reporting NK and T cell functional deficiencies in people living with AIDS (3) or in MSM who were asymptomatic and not known to have an HIV-1 infection at the time of the study (4). These early studies pointed out or confirmed that the CD4^+^ T cell was a major target of the new disease eventually called AIDS and that loss of CD4^+^ T cells or their function could begin to explain AIDS-related opportunistic infections and malignancies.

As an understanding of the clinical and laboratory details of immunodeficiency in AIDS evolved, it became important to understand what cells were infected by this new virus, first reported in 1983 (5). In 1986, Ho, Rota, and Hirsh reported the ability of HTLV-III (subsequently named HIV-1) to infect monocyte lineage cells (6). Later, van Noesel and colleagues in Amsterdam demonstrated evidence for selective loss of memory T cells in men living with HIV-1 (7), and Spina, Prince, and Richman subsequently demonstrated infection of CD45RO^+^ memory T cells by HIV-1 and described functional deficiencies of TCR triggering in memory T cells (8). Thus, by the mid-1990s, papers in the *JCI* had established or confirmed that the major targets of HIV-1 were CD4^+^ T cells and monocytes, and that HIV-1 infection was indeed associated with cellular functional deficiencies, particularly in helper CD4^+^ T cells. CD4^+^ T helper dysfunction was defined in these and other reports as resulting in profound effects on populations of memory T cells (6, 7) and, importantly, on B cell responses to specific antigens (2).

## The origin of immune cells that return after ART

As combinations of medications termed antiretroviral treatment (or therapy) (ART) began to be used to treat HIV-1 infection in the mid-1990s, patients began showing rises in CD4^+^, CD45RO^+^ memory T cells. In 1999, Haynes and team demonstrated that HIV-1–infected patients with myasthenia gravis who underwent thymus removal still experienced peripheral CD4^+^ T cell rises following ART, demonstrating that peripheral T cells were primarily responsible for blood T cell reconstitution in those individuals (9). The same year, Bucy et al. directly demonstrated that the initial increase in blood CD4^+^ T cells after ART was due to redistribution of T cells from peripheral lymphoid tissues (10). They also showed that T cell activation by HIV-1 resulted in elevated T cell adhesion molecules responsible for T cell migration to tissues, and that ART markedly reduced T cell adhesion molecule expression (10). Subsequently, Altfeld et al. reported that, as ART was terminated in chronically HIV-1–infected individuals, the rise in blood CD8^+^ T cells was due to resurgent viral stimulation of preexisting CD8^+^ T cells in lymph nodes (11).

In 2000, McCune et al. studied T cell turnover in HIV-1 seropositive patients and found that a shortened half-life of CD4^+^ T cells without a compensatory increase in CD4^+^ T cell production provided one explanation for the AIDS-associated decrease in CD4^+^ T cells (12). Hellerstein et al. went on to demonstrate that the greatest impact of HIV-1 infection was a decrease in the induction of long-lived progenitor T cells (13). This work challenged the suggestion that the primary cause of CD4^+^ T cell loss was due to direct killing by HIV-1. It is now understood that most of the increased proliferation and accelerated T cell death observed in AIDS results from massive bystander T cell activation (13). Thus, the increased levels of proliferation of CD4^+^ and CD8^+^ T cells in HIV-1 infection are not primarily related to a homeostatic mechanism to compensate for loss of CD4^+^ T cells directly killed by HIV-1, but rather are due to extensive and chronic viral-induced immune cell activation followed by bystander CD4^+^ and CD8^+^ T cell apoptosis (13)—a fundamental aspect of the associated immunodeficiency.

## Establishment of the latent reservoir of HIV-1

An important and continued area of interest in HIV-1 pathogenesis associated with seminal observations in the *JCI* over years is that of HIV persistence and latency. McElrath, Steinman, and Cohn reported in 1991, that latent HIV-1 infection is present in less than 1% of blood monocytes and T cells in patients seropositive for HIV (14). Shortly thereafter, Mikovits and team explored the release of latent HIV-1 from monocytes following T cell activation (15). In 2000, children living with HIV-1 were shown to have a stable provirus reservoir in resting CD4^+^ T cells (16). In the same year, Zhang, et al. examined individuals infected with HIV-1 who had complete suppression by ART. Notably, viral rebound after cessation of therapy likely originated from activation of virus from the latent reservoir (17). In contrast, viral rebound in patients with incomplete suppression of virus by ART was likely triggered by ongoing low-level replication of HIV-1 (18). In 2005, Chun and team made a surprising observation. During long-term ART, HIV-1–infected individuals continually replenished their viral reservoir through ongoing replication and have substantially higher levels of HIV proviral DNA in activated CD4^+^ T cells when compared with resting CD4^+^ T cells (17). It is now recognized that both ongoing low level replication as well as activation of latent virus can contribute to viral rebound.

## Maintenance of the latent virus pool over time

Several papers published in *JCI* have markedly enhanced our understanding of HIV reservoirs and informed HIV-1 cure strategies. In 2017, Lee et al. found that Th1 CD4^+^ T cells were relatively enriched for intact replication-competent versus defective proviruses (19) and this population was likely maintained over time via clonal expansion. Duette et al. found that CD4^+^ T effector memory cells had higher levels of intact proviral sequences over time compared with other CD4^+^ populations. Further, escape mutations remained stable within this CD4^+^ population, and expression of the HIV accessory gene Nef, from intact and defective proviruses, could promote immune evasion, thus protecting infected CD4^+^ T cells from CD8^+^ T cell killing via downregulation of HLA class I antigens on CD4^+^ T cells (20).

Considerable work has been published in the *JCI* on the origin of virus that is nonsuppressible by ART in spite of adherence to ART and in the absence of viral drug resistance. Halvas et al. reported in 2020 that HIV-1 viremia that is not suppressible by ART, even with switching or intensifying medications, can originate from proliferating infected T cell clones (21). Most recently, White et al. reported that nonsuppressed viremia occurs in about one in 205 persons living with HIV-1 (PLWH) on ART (22). Further, in a subset of PLWH, 5′-leader virus mutations gave rise to nonsuppressible viremia with non-infectious virus that did not express envelope and thereby could not spread (22). These findings were of interest because HIV-1 replication requires the 5′-leader genomic region; however, it remains unclear why the immune system cannot clear infected CD4^+^ T cells with these types of defective proviruses (23). If successful immune responses depend on Env-binding antibodies and antibody-dependent cellular cytotoxicity (ADCC), then 5′-leader mutational escape serves as an escape mechanism from immune clearance of virus-infected cells (23). A deeper understanding of nonlymphoid tissue reservoirs are relevant to cure strategies, as demonstrated by Tang and authors who revealed an inducible and replication-competent virus was harbored in cells isolated from the brains of nonhuman primates and people on ART (24).

**Figure 1:**
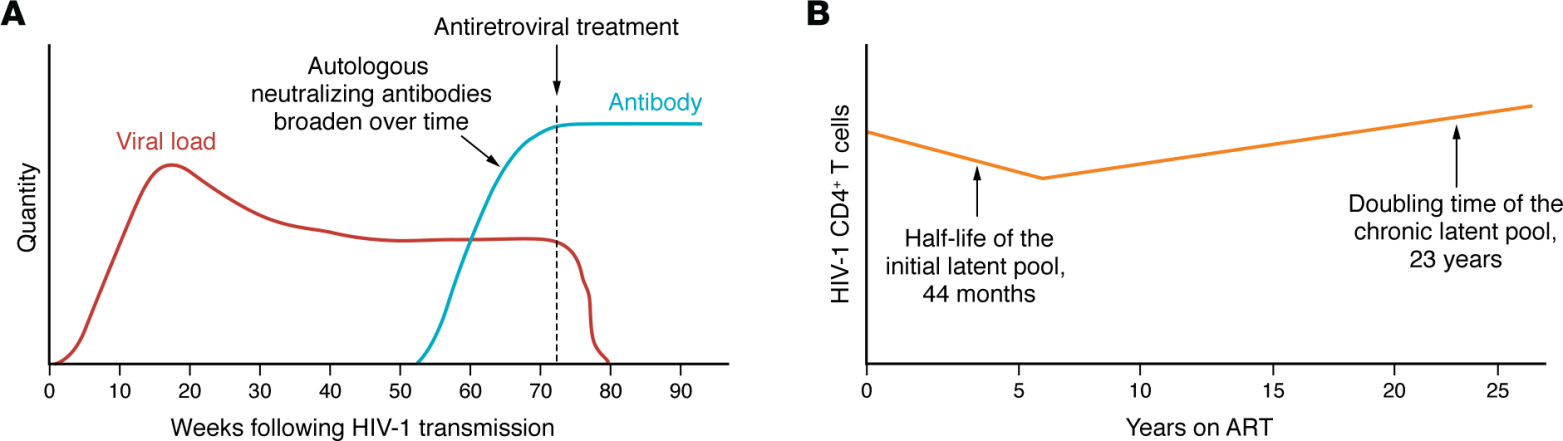
The latent pool of HIV-1 CD4^^+^^ T cells is established in the weeks and years following HIV-1 transmission. (**A**) Autologous neutralizing antibodies rise following untreated HIV-1 infection. After approximately 60 days, sufficient viral replication has occurred to stimulate autologous neutralizing antibodies. Following induction, the antibodies can develop breadth and potency of neutralization (29). (**B**) HIV-1 infection over a 25-year timecourse on ART. The half-life of the initial latent pool of CD4^^+^^ T cells is approximately 44 months. After long duration of antiretroviral treatment, the latent pool increases with a doubling time of approximately 23 years due to proliferation of infected CD4^^+^^ T cells (25).

More recently in 2023, McMyn et al. in the laboratory of Janet Siliciano reported that the latent reservoir of inducible, infectious HIV-1 in individuals on ART does not decease despite viremia suppression over decades (25). Rather, the virus reservoir decay slows over time on ART and may even reverse in PLWH after very long-term ART. The authors went on to show that proliferation of infected CD4^+^ T cells is likely a primary factor in stability of this reservoir of replication-competent virus (Figure 1). These results have important implications for clinical management of PLWH as they directly demonstrate the need for lifelong ART. Importantly, the HIV-1 reservoir of infected CD4^+^ T cells being maintained over two decades continues to be a major barrier to a HIV-1 cure (25). However, in a small number of PLWH, a strategy for a functional cure has been raised. A recent study of viral reservoirs in individuals that contain the virus for years in the absence of antiretrovirals provided insights into endogenous mechanisms of control that could impact functional cure strategies (26). Persistent controllers (PCs) over many years had lower ratios of intact to defective viral ratios compared with those that eventually lost control, termed transient controllers (TCs). Clonally expanded intact provirus was only found in the PC group and the provirus in the PC group was uniquely located in centromeric satellite DNA or zinc finger genes (26).

HIV-1–host interactions remain a critical area of cure-AIDS research. Anti-PD1 CAR-T cells infused in SIV-infected rhesus macaques efficiently eliminated follicular helper T cells, thus decreasing viral load (27). However, these salutary effects were counter balanced by long term CAR-T immunosuppression (27). Thus, therapies that balance selective targeting of HIV-1–infected CD4^+^ cells by the host immune system without suppression of protective immune responses may have promise in decreasing or eliminating the latent pool of HIV-1–infected cells.

Finally, the induction of autologous neutralizing antibodies represents one host immune response potentially capable of targeting the infected pool of CD4^+^ T cells (28). Approximately 60 days of HIV-1 infection defines the threshold for the induction of autologous neutralizing antibodies, following which there is an increase in antibody potency and breadth (29) (Figure 1). In depth analysis of the host response in experimental HIV-1 vaccination trials holds the promise of elucidating modes of vaccination for boosting autologous T and B cell responses that may aid in control of HIV-1. Moreover, based on the remarkable impact HIV-1 research has had on enabling the rapid response to other pathogens such as SARS-CoV-2, we can expect that HIV-1 pathogenesis, prevention, and cure research will continue to enhance impactful research in multiple disease categories in the future.

## Supplementary Material

Supplemental data

## Version 1 07/01/2024

Electronic publication

## References

[1] Landay A (1983). Immunologic studies in asymptomatic hemophilia patients. Relationship to acquired immune deficiency syndrome (AIDS). J Clin Invest.

[2] Nicholson JK (1984). Immunoregulatory subsets of the T helper and T suppressor cell populations in homosexual men with chronic unexplained lymphadenopathy. J Clin Invest.

[3] Rook AH (1983). Interleukin-2 enhances the depressed natural killer and cytomegalovirus-specific cytotoxic activities of lymphocytes from patients with the acquired immune deficiency syndrome. J Clin Invest.

[4] Shearer GM (1984). Functional T lymphocyte immune deficiency in a population of homosexual men who do not exhibit symptoms of acquired immune deficiency syndrome. J Clin Invest.

[5] Barre-Sinoussi F (1983). Isolation of a T-lymphotropic retrovirus from a patient at risk for acquired immune deficiency syndrome (AIDS). Science.

[6] Ho DD (1986). Infection of monocyte/macrophages by human T lymphotropic virus type III. J Clin Invest.

[7] van Noesel CJ (1990). Functional and phenotypic evidence for a selective loss of memory T cells in asymptomatic human immunodeficiency virus-infected men. J Clin Invest.

[8] Spina CA (1997). Preferential replication of HIV-1 in the CD45RO memory cell subset of primary CD4 lymphocytes in vitro. J Clin Invest.

[9] Haynes BF (1999). Analysis of the adult thymus in reconstitution of T lymphocytes in HIV-1 infection. J Clin Invest.

[10] Bucy RP (1999). Initial increase in blood CD4(+) lymphocytes after HIV antiretroviral therapy reflects redistribution from lymphoid tissues. J Clin Invest.

[11] Altfeld M (2002). Expansion of pre-existing, lymph node-localized CD8+ T cells during supervised treatment interruptions in chronic HIV-1 infection. J Clin Invest.

[12] McCune JM (2000). Factors influencing T-cell turnover in HIV-1-seropositive patients. J Clin Invest.

[13] Hellerstein MK (2003). Subpopulations of long-lived and short-lived T cells in advanced HIV-1 infection. J Clin Invest.

[14] McElrath MJ (1991). Latent HIV-1 infection in enriched populations of blood monocytes and T cells from seropositive patients. J Clin Invest.

[15] Mikovits JA (1992). Activation of infectious virus from latent human immunodeficiency virus infection of monocytes in vivo. J Clin Invest.

[16] Persaud D (2000). A stable latent reservoir for HIV-1 in resting CD4(+) T lymphocytes in infected children. J Clin Invest.

[17] Chun TW (2005). HIV-infected individuals receiving effective antiviral therapy for extended periods of time continually replenish their viral reservoir. J Clin Invest.

[18] Zhang L (2000). Genetic characterization of rebounding HIV-1 after cessation of highly active antiretroviral therapy. J Clin Invest.

[19] Lee GQ (2017). Clonal expansion of genome-intact HIV-1 in functionally polarized Th1 CD4+ T cells. J Clin Invest.

[20] Duette G (2022). The HIV-1 proviral landscape reveals that Nef contributes to HIV-1 persistence in effector memory CD4+ T cells. J Clin Invest.

[21] Halvas EK (2020). HIV-1 viremia not suppressible by antiretroviral therapy can originate from large T cell clones producing infectious virus. J Clin Invest.

[22] White JA (2023). Clonally expanded HIV-1 proviruses with 5′-leader defects can give rise to nonsuppressible residual viremia. J Clin Invest.

[23] Emery A (2023). Nonsuppressible viremia during HIV-1 therapy meets molecular virology. J Clin Invest.

[24] Tang Y (2023). Brain microglia serve as a persistent HIV reservoir despite durable antiretroviral therapy. J Clin Invest.

[25] McMyn NF (2023). The latent reservoir of inducible, infectious HIV-1 does not decrease despite decades of antiretroviral therapy. J Clin Invest.

[26] Gasca-Capote C (2024). The HIV-1 reservoir landscape in persistent elite controllers and transient elite controllers. J Clin Invest.

[27] Eichholz K (2024). Anti-PD-1 chimeric antigen receptor T cells efficiently target SIV-infected CD4+ T cells in germinal centers. J Clin Invest.

[28] Bertagnolli LN (2020). Autologous IgG antibodies block outgrowth of a substantial but variable fraction of viruses in the latent reservoir for HIV-1. Proc Natl Acad Sci U S A.

[29] Whitehill GD (2024). Autologous neutralizing antibody responses after antiretroviral therapy in acute and early HIV-1. J Clin Invest.

