# Supplementary material for: The other pandemic: lessons from 40 years of HIV research
Source: J Clin Invest. 2024 Jul 1;134(13):e183039. doi: 10.1172/JCI183039 (PMC11213503; doi:10.1172/JCI183039)
Supplement: Supplemental data [file jci-134-183039-s241.pdf]

List of Patent Applications or Issued Patents relating to HIV Vaccine Development

BISPECIFIC MOLECULES COMPRISING AN HIV-1 ENVELOPE TARGETING ARM	US 10,717,778
BISPECIFIC MOLECULES COMPRISING AN HIV-1 ENVELOPE TARGETING ARM	US 16/890,380
MOSIAC HIV ENVELOPE IMMUNOGENIC POLYPEPTIDES	US 9,855,329
HIV-1 NEUTRALIZING ANTIBODIES AND USES THEREOF	US 11,071,783
HIV-1 NEUTRALIZING ANTIBODIES AND USES THEREOF	US 11,944,681
HIV-1 NEUTRALIZING ANTIBODIES AND USES THEREOF (V3 ANTIBODIES)	US 10,344,077
HIV-1 NEUTRALIZING ANTIBODIES AND USES THEREOF (CD4bs antibodies)	US 10,450,368
HIV ENVELOPES TO INDUCE CH235 LINEAGE ANTIBODIES	US 10,968,255
HIV ENVELOPES TO INDUCE CH235 LINEAGE ANTIBODIES	US 11,884,704
COMPOSITIONS AND METHODS FOR INDUCING HIV-1 ANTIBODIES	US 11,246,920
COMPOSITIONS AND METHODS FOR INDUCING HIV-1 ANTIBODIES	US 11,318,197
COMPOSITIONS COMPRISING MODIFIED HIV ENVELOPES	US 11,814,413
MOSAIC HIV-1 ENVELOPES TO INDUCE ADCC RESPONSES	US 11,773,144
A METHOD OF INHIBITING HIV INFECTION	US 6,432,405
HIV-1 ENVELOPE STABILIZING MUTATIONS	US 17/281,918 EP 19868740.2 CA 3,114,870
HIV ENVELOPES TO INDUCE HIV-1 ANTIBODIES	US 17/281,933 EP 19868959.8 CA 3,115,232
COMPOSITIONS COMPRISING V2 OPT HIV ENVELOPES	US 17/771,349 CA 3,234,955
COMPOSITIONS COMPRISING V2 OPT HIV ENVELOPES	PCT/US2024/024154
COMPOSITIONS COMPRISING V2 OPT HIV ENVELOPES	US 18/700,679
HIV ENVELOPES TO INDUCE HIV-1 ANTIBODIES	US 18/032,497 EP 21883746.6 CA 3,196,100
HIV-1 ENVELOPE GLYCOPEPTIDE NANOPARTICLES AND THEIR USES	US 18/280,935 EP 22767805.9
HIV-1 ENVELOPE GLYCOPEPTIDE NANOPARTICLES AND THEIR USES	CA 3,211,186
COMPOSITIONS COMPRISING HIV ENVELOPES TO INDUCE HIV ANTIBODIES	US 18/700,677
HIV VACCINE IMMUNOGENS FOR V3-GL YCAN TARGETING ANTIBODIES	PCT/US2023/073257
STABILIZATION OF HUMAN IMMUNODEFICIENCY VIRUS (HIV) ENVELOPES	PCT/US2023/077611
MODELING AND STABILIZING HIV ENVELOPES	PCT/US2024/016309

COMPOSITIONS COMPRISING HIV-1 ENVELOPES WITH ENGINEERED V1V2 OR MRNAs ENCODING THE SAME FOR BROAD V3-GLYCAN NEUTRALIZING ANTIBODY BINDING PCT/US2023/077804

COMPOSITIONS COMPRISING HIV-1 ENVELOPES WITH ENGINEERED V1V2 OR MRNAs ENCODING THE SAME FOR BROAD V3-GLYCAN NEUTRALIZING ANTIBODY BINDING PCT/US2023/077808

COMPOSITIONS COMPRISING mRNAs ENCODING HIV-1 MEMBRANE PROXIMAL EXTERNAL REGION (MPER) PEPTIDES PCT/US2023/077677

COMPOSITIONS COMPRISING mRNAs ENCODING HIV-1 MEMBRANE PROXIMAL EXTERNAL REGION (MPER) PEPTIDES PCT/US2023/077686

HIV-1 FUSION PEPTIDE NANOPARTICLE VACCINES US 63/540,484

HIV-1 ENVELOPES FOR V3-GLYCAN NEUTRALIZING ANTIBODY BINDING US 63/634,216

HIV-1 ENV IMMUNOGENS THAT TARGET UCAS AGAINST MULTIPLE ANTIGENIC SITES US 63/634,195

A COMPUTATIONAL PROCESS FOR IDENTIFYING HIV-1 BROADLY NEUTRALIZING ANTIBODIES, THEIR PRECURSORS, AND INTERACTIVE IMMUNOGENS US 63/634,200

BOOSTING BnAbs AGAINST HIV-1 US 63/634,232
